# Amyand’s Hernia

**DOI:** 10.5334/jbr-btr.1402

**Published:** 2018-01-05

**Authors:** Wouter Mebis, Pieter Hoste, Tjeerd Jager

**Affiliations:** 1 Algemeen Stedelijk Ziekenhuis Aalst, BE

**Keywords:** Amyand’s hernia, Appendicitis, Inguinal, Ultrasound, Computed tomography

## Case

A 64-year-old man consulted the emergency department with intermittent abdominal pain during the past 10 days. Clinical examination demonstrated tenderness in the right iliac fossa towards the inguinal region. Blood testing showed no signs of inflammation.

**Figure 1:**
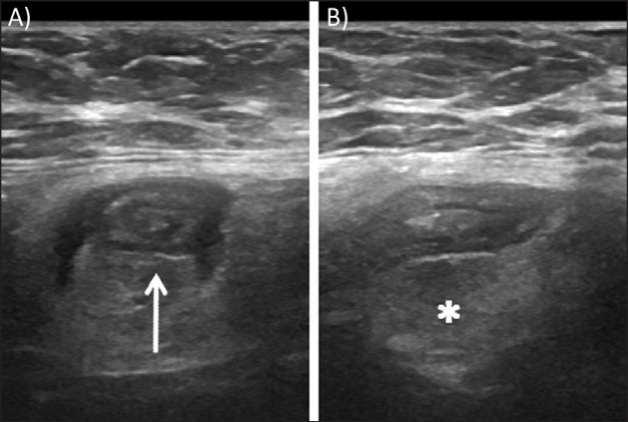
Grey-scale ultrasound. Axial **(A)** and sagittal views of the appendix within an inguinal hernia sac **(B)**.

Ultrasound (US) examination of the abdomen revealed a direct inguinal hernia protruding anteromedially and inferiorly to the inferior epigastric vessels. A small tubular structure compatible with the appendix was present within the hernia sac (Figure 1, arrow). The appendix diameter was at the upper normal range, and the surrounding fat was hyperechoic (asterisk). Consequently, the patient was preliminary diagnosed with Amyand’s hernia with signs of inflammation.

**Figure 2:**
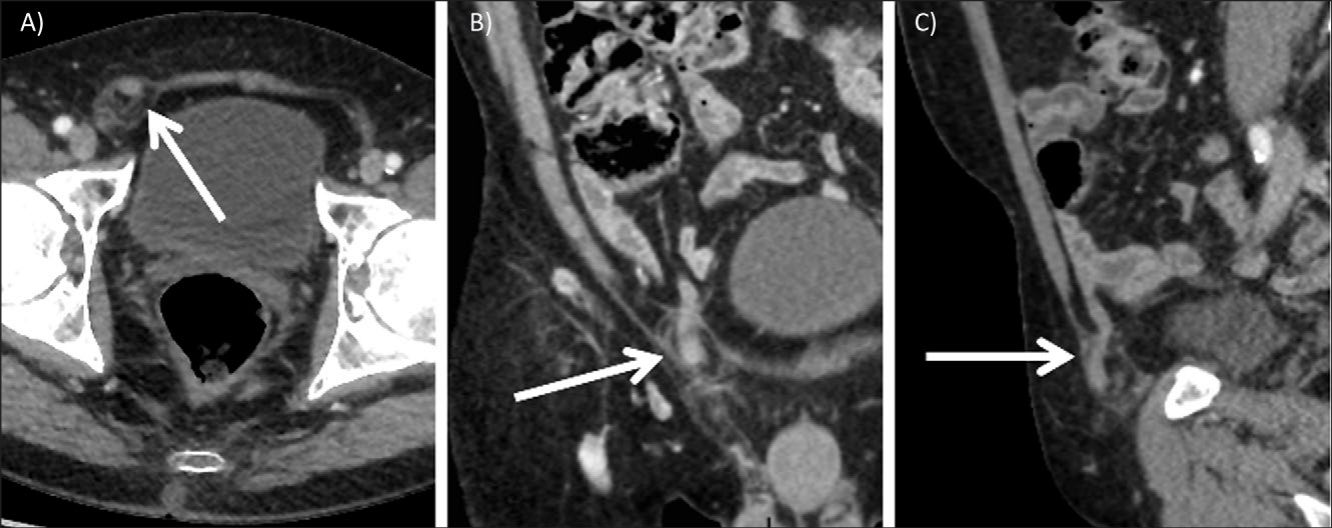
Contrast-enhanced computed tomography of the abdomen. Axial **(A)**, coronal **(B)** and sagittal **(C)** views of the appendix located within the right inguinal hernia sac.

Complementary computed tomography (CT) of the abdomen confirmed Amyand’s hernia (arrows, Figure 2). Whether the inflammation was caused by some degree of incarceration or as a result of acute appendicitis remained unclear. There were no signs of complications such as perforation or abscess.

## Comment

Amyand’s hernia is a rare type of inguinal hernia where the appendix is located within and/or incarcerated in the hernia sac. This is the case in about 1% of all hernias, and it occurs more frequently in children because of the patent processus vaginalis. Amyand’s hernias can be subdivided into two types: direct or indirect inguinal hernias. The appendix may remain perfectly normal but could become inflamed with subsequent perforation and abscess if diagnosis is delayed. However, the incidence of appendicitis within an inguinal hernia is very rare (about 0.1% of all inguinal hernias). Clinical symptoms can be misleading and more often resemble those of a (strangulated) inguinal hernia than the classic signs and symptoms of appendicitis. Sometimes a palpable inguinal mass may be present [1].

Ultrasound is an excellent technique to evaluate the inguinal region and can be used to detect all types of inguinal hernias, including Amyand’s hernia. US is safe and cheap, but remains operator-dependent.

Computed tomography (CT) can be used to confirm the diagnosis if necessary. At the same time, possible intra-abdominal complications like perforation and abscess can be ruled out, especially in a preoperative setting [1].

When diagnosis remains unclear, surgery can both be diagnostic and therapeutic. Treatment depends on the surgeon’s personal choice. Removing a healthy appendix is subject to a medical debate which has not reached a consensus yet [1].
